# Pulmonary fibrosis *in vivo* displays increased p21 expression reduced by 5-HT_2B_ receptor antagonists *in vitro* – a potential pathway affecting proliferation

**DOI:** 10.1038/s41598-018-20430-0

**Published:** 2018-01-31

**Authors:** Anna Löfdahl, Kristina Rydell-Törmänen, Anna-Karin Larsson-Callerfelt, Christina Wenglén, Gunilla Westergren-Thorsson

**Affiliations:** 1 Lung Biology, Department of Experimental Medical Science, Lund University, Lund, Sweden; 2 AnaMar AB, Lund, Sweden

## Abstract

Serotonin (5-hydroxytryptamine) has repeatedly been associated with the development of fibrotic disorders such as pulmonary fibrosis. By blocking the binding of 5-HT to 5-HT_2B_ receptors with receptor antagonists, several pro-fibrotic mechanisms can be inhibited. Bleomycin-induced pulmonary fibrosis is a model used to evaluate pathological mechanisms and pharmacological interventions. Previously we have shown attenuated fibrosis in systemic bleomycin-treated mice following treatment with two 5-HT_2B_ receptor antagonists (EXT5 and EXT9). Our aim is to further identify cellular effects and signaling pathways associated with the anti-fibrotic effects of EXT5/9. Gene expressions in lung tissues from systemic bleomycin-treated mice were examined, revealing significant increased expression of *Cdkn1α* (a gene coding for p21), particularly in distal regions of the lung. *In vitro* studies in human lung fibroblasts revealed increased levels of p21 (p = 0.0032) and pAkt (p = 0.12) following treatment with 5-HT (10 µM). The induction of p21 and pAkt appears to be regulated by 5-HT_2B_ receptors, with diminished protein levels following EXT9-treatment (p21 p = 0.0024, pAkt p = 0.15). Additionally, 5-HT induced fibroblast proliferation, an event significantly reduced by EXT5 (10 µM) and EXT9 (10 µM). In conclusion, our results suggest that 5-HT_2B_ receptor antagonism attenuates pulmonary fibrosis in part by anti-proliferative effects, associated with inhibited pAkt/p21 signaling pathway.

## Introduction

Idiopathic pulmonary fibrosis (IPF) is a devastating interstitial lung disease with unknown etiology and limited treatment options^1^. The pathogenesis of IPF resides in ongoing and aberrant chronic wound repair responses. Realistic models of this disease are lacking, but the anti-tumor drug, bleomycin (BLM) is widely used to model pulmonary fibrosis *in vivo* via subcutaneous (s.c.), i.e. systemic administration, or more commonly through an intratracheal route of administration^2,3^. We have previously shown that following systemic BLM administrations, animals display increased accumulation of collagens, extracellular matrix (ECM) proteins and collagen producing cells^3,4^, mimicking numerous pathological mechanisms associated in IPF^5^. In response to lung injury and tissue repair, several molecular mechanisms are triggered such as proliferation of fibroblasts and the formation of collagen-producing myofibroblasts^6^, resulting in selective gene expressions. The formation of collagens correlates with upregulation of genes involved in cell proliferation^7^, underlining the important interplay between ECM deposition and cell proliferation. Furthermore, in BLM-treated mice, pulmonary levels of serotonin (5-hydroxytryptamine, 5-HT) and expression of 5-HT_2_ receptors are elevated^8,9^, which is believed to be associated with increased myofibroblast differentiation and matrix deposition. This is supported by findings showing that treatment with selective 5-HT_2_ receptor antagonists inhibit BLM-induced tissue remodeling^4,10^.

Several important cellular responses are mediated via 5-HT_2_ receptors. In human endothelial cells, 5-HT has been shown to trigger an increased expression of Akt, a serine/threonine protein kinase involved in signaling pathways regulating migration, survival and proliferation^11^. Phosphorylated Akt (pAkt) and 5-HT as well as the expression of the 5-HT_2B_ receptor were increased in lungs of mice following orotracheal administered BLM^8^. One phosphorylation substrate for pAkt is p21^12^, a protein elevated at cellular damage and in pulmonary fibrosis^13–16^. Akt also promotes cell cycling by regulating substrates such as p21 and cyclin D1. Akt-induced p21 can promote the assembly and activation of cyclin D1-Cdk4 complex, triggering G1/S transition^17,18^. 5-HT modulates many important regulatory functions, and a recent study demonstrates increased cell proliferation and DNA synthesis in hepatocytes following activation of 5- HT_2B_ receptors^19^.

We recently described how a preventive treatment with 5-HT_2B_ receptor antagonists EXT5 and EXT9 inhibited several pulmonary fibrotic events in BLM-treated mice, such as reduced numbers of pulmonary myofibroblasts and collagen producing cells as well as an overall reduced pulmonary tissue density^4^. In the present study, we continued to investigate the effect of EXT5 and EXT9, focusing on pulmonary gene expressions in BLM-treated mice, suggestive of signaling pathways associated with cell proliferation. The effects of the 5-HT_2B_ receptor antagonists on proliferation and related intracellular signaling proteins were further studied in human *in vitro* cultures, relating to our earlier anti-fibrotic findings *in vivo*^4^.

## Results

### Gene for p21 upregulated in BLM-treated mice

**Figure 1:**
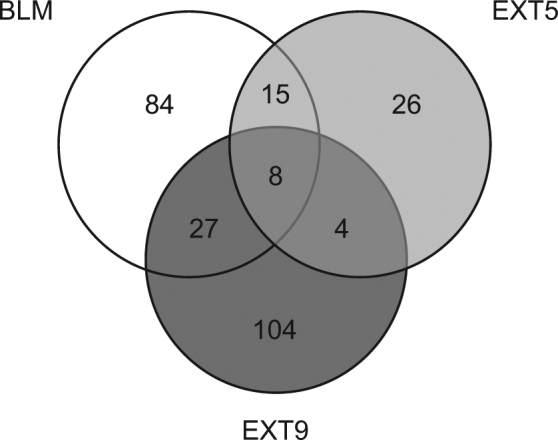
Venn diagram of statistically significant differentially expressed genes in BLM-, EXT5 and EXT9-treated mice. Distal lung tissue from mice subjected to s.c. administrations of BLM in combination with p.o. treatment with EXT5, EXT9 or saline was isolated for RNA. With whole genome gene expression array and SAM analysis, significant differentially expressed genes were identified, in comparison to healthy saline controls. p < 0.05, q = 0. N = 6 (N = number of animals analyzed).

**Figure 2:**
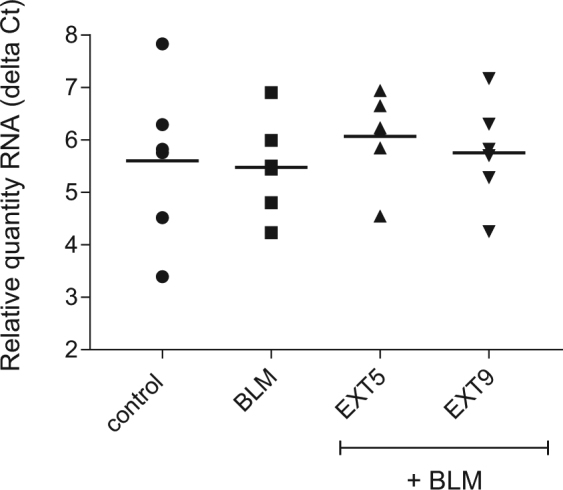
Lung tissue expression of *Cdkn1α* in pulmonary fibrotic mice. Gene expression levels of *Cdkn1α* in lung tissue from control animals, BLM- and EXT5- and EXT9-treated mice were analyzed with rt-qPCR. The relative expressions of *Cdkn1α*, normalized to reference gene *GAPDH*, are presented as delta Ct values for each animal with group mean (n = 6). Statistical analysis was performed with one-way ANOVA.

We have previously examined initial events in pulmonary fibrosis *in vivo*, utilizing a formerly described model with repeated s.c. administrations of BLM – a systemic model demonstrating early alterations in ECM lung compositions and cell turnover^3,4^. Whole genome gene expression in distal lung tissue was analyzed after two weeks of BLM-treatment. Eight probes, coding for six genes, were significantly differentially expressed in BLM-treated mice, in comparison to control animals (Fig. 1). The expression of *Cdkn1α* was significantly upregulated (p < 0.05, q = 0) in BLM-treated mice, recognized by multiple significant probes for *Cdkn1α* (Table 1). *Cdkn1α* codes for p21, a protein associated with cell cycle regulation and cell damage^15^. Treatment with EXT9, and to some extent EXT5, showed a modest reduction in *Cdkn1α expression*, however the fold change (FC) of the probes was not proven as statistical significant, in comparison to BLM-treated mice. Two weeks of systemic administrations of BLM generated a mild to moderate model of pulmonary fibrosis, thus accompanied with small variation in disease related gene expressions. Analysis of lung tissue samples (examining larger parts of the lung) with rt-qPCR, demonstrated small differences in levels of *Cdkn1α* between treatment groups with some expression variability within groups (Fig. 2). In BLM-treated mice a minor increase of *Cdkn1α* (ΔCt = 5.48, FC = 1.1) was detected, in comparison to control animals (ΔCt = 5.60, FC = 1). Treatment with EXT5 or EXT9 showed tendencies in reducing the expression (EXT5 ΔCt = 6.07, FC = 0.7; EXT9 ΔCt = 5.75, FC = 0.9), however, not proven as statistical significant (Fig. 2).

**Table 1:** Gene expression in distal lung tissue from BLM-treated mice.

Fold change			
Gene symbol	BLM	EXT5	EXT9
Cdkn1a	2.73	2.25	2.10
Gdf15	1.55	1.52	1.51
Prc1	1.56	1.67	1.65
Cdkn1a	2.04	2.00	1.51
Cdkn1a	1.88	1.82	1.50
Phlda3	1.47	1.62	1.24
Igfbp2	1.85	1.64	1.71
LOC665235	−1.38	−1.30	−1.43

Significant differentially expressed gene probes (q = 0) in BLM-, EXT5- and EXT9-treated mice, in comparison to saline controls. Probe expression levels shown as fold change of gene symbol, with *Cdkn1α* identified with three different probes.

### Reduced proliferation of lung fibroblasts

**Figure 3:**
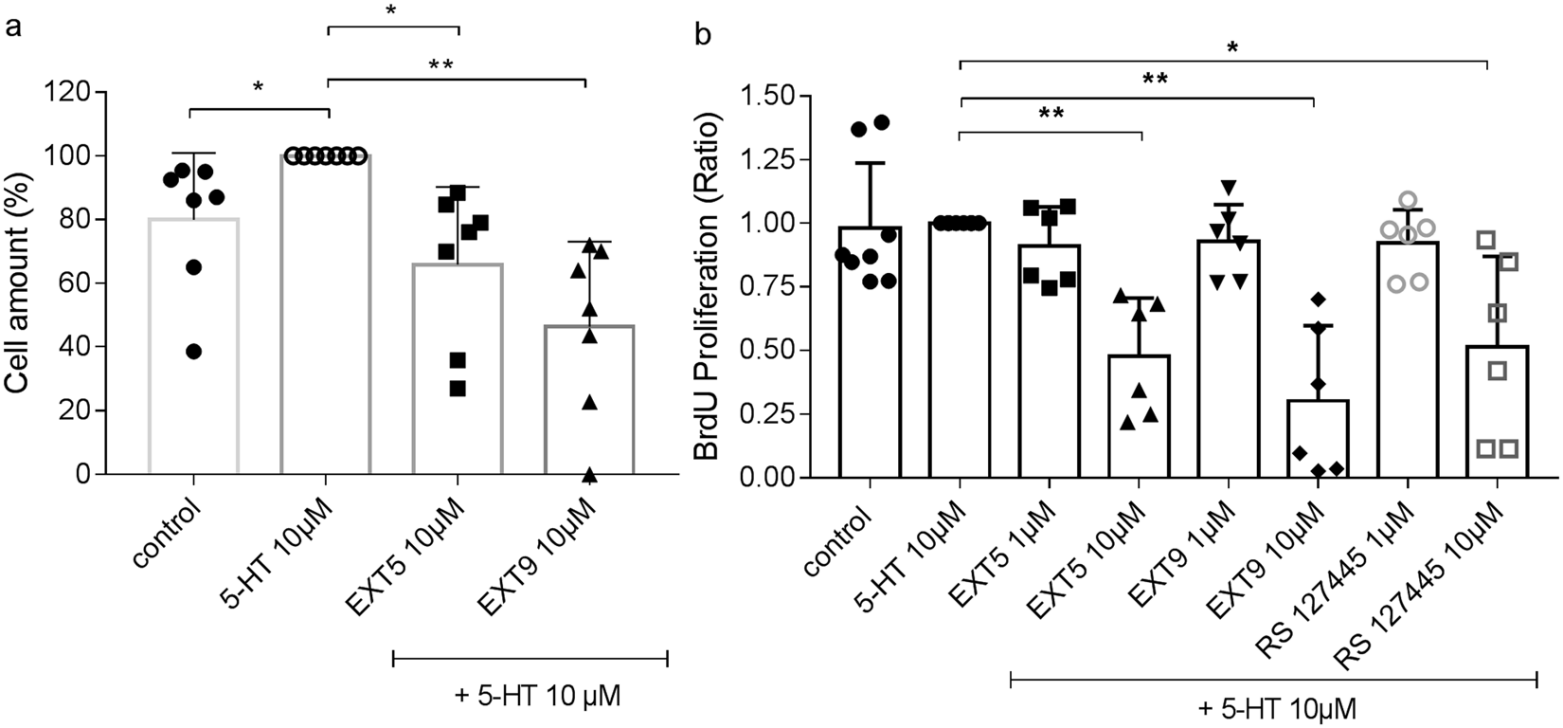
Proliferation of HFL-1 cells treated with 5-HT and 5-HT_2B_ receptor antagonists. Cells were treated with 5-HT (10 µM), without (**a**) or with 1 h pre-treatment with 5-HT_2B_ receptor antagonists EXT5, EXT9 or RS 127445 (1 µM, 10 µM). Cell amount was measured after 48 h with crystal violet incorporation (**a**) and relative amount proliferating cells was quantified with BrdU incorporation (**b**). Untreated cells served as control (0.1% DMSO). Results are represented as individual values with mean + s.d. of a minimum of six independent experiments (n = 6–8), performed with one sample t-test for statistical analysis.

We have previously shown that collagen producing cells i.e. fibroblasts and myofibroblasts were increased in mice treated s.c. with BLM, a common pulmonary feature recognized in patient with fibrosis^20^. An important regulator of cell cycle progression is p21, and activation of the 5-HT_2B_ receptor has shown to induce cellular growth in 5-HT_2B_ receptor-transfected murine fibroblasts^21^. To provide evidence supporting a 5-HT_2B_ receptor mediated cell cycle regulation in human cell cultures, proliferation of HFL-1 cells was studied following treatment with 5-HT in combination with or without 5-HT_2B_ receptor antagonists; EXT5, EXT9 and RS 127445 (1 µM, 10 µM). The examination of crystal violet incorporation in HFL-1 cells showed a reduction in total amount of cells following 48 h treatment with EXT5 (10 µM) (65.8 ± 24%, p = 0.0101) or EXT9 (10 µM) (46.4 ± 27%, p = 0.0018), in comparison to 5-HT alone (100%) (Fig. 3a). Furthermore, the total amount dividing cells (BrdU proliferation) was also reduced to 48% (p = 0.0025), 31% (p = 0.0021) and 52% (p = 0.0203) with 10 µM of EXT5, EXT9 and RS 127445, respectively (Fig. 3b), supporting our results on total cell amount.

### 5-HT_2B_ receptor antagonists affect cell cycle progression

**Figure 4:**
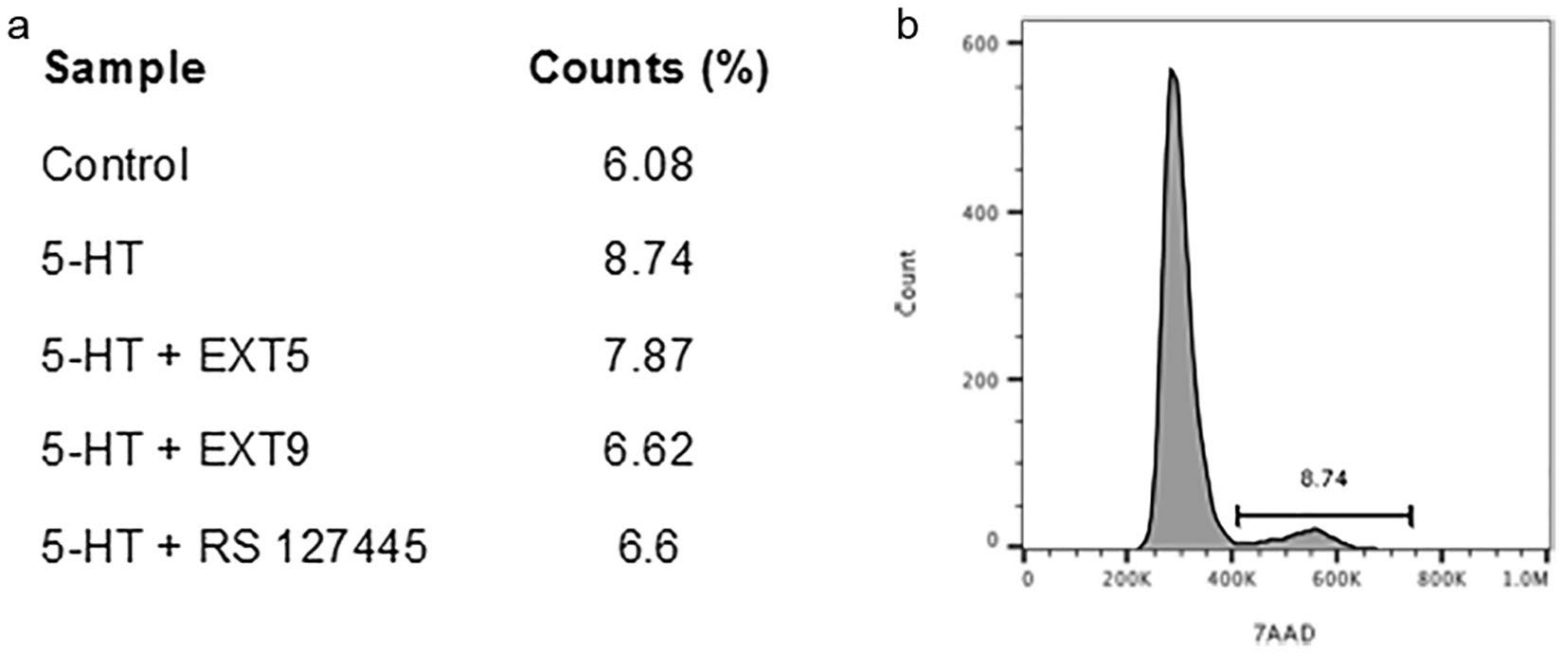
Cell cycle regulation of HFL-1 cells by 5-HT and 5-HT_2B_ receptor antagonists. HFL-1 cells were treated with 5-HT (10 µM) in combination with or without receptor antagonists EXT5, EXT9 and RS 127445 (10 µM). After 48 h, cells were stained with 7AAD and total amount of cycling cells was analyzed with flow cytometry, identifying total amount of cycling cells in the S/G2/M phase (**a**). A representative image demonstrating the distinct cell cycle peaks and gating for S/G2/M is presented with specified percentage cell cycle counts (8.74% of sample containing 5-HT-treated cells) (**b**).

To further validate the role of 5-HT and 5-HT_2B_ receptors in regulating cell cycle progression, HFL-1 cells were treated with 5-HT (10 µM) in combination with or without 5-HT_2B_ receptor antagonists EXT5 (10 µM), EXT9 (10 µM) and RS 127445 (10 µM), and analyzed with flow cytometry for the detection and quantification of cells in S/G2/M phase (i.e. dividing cells). Results showed an increased amount of cells in S/G2/M phase following treatment with 5-HT (8.74%) in comparison to control (6.08%) (Fig. 4a,b). Treatment with receptor antagonists reduced number of dividing cells; EXT5 (7.87%), EXT9 (6.62% and RS 127445 (6.60%) (Fig. 4a).

### Undetected cell toxicity with 5-HT_2B_ receptor antagonists

**Figure 5:**
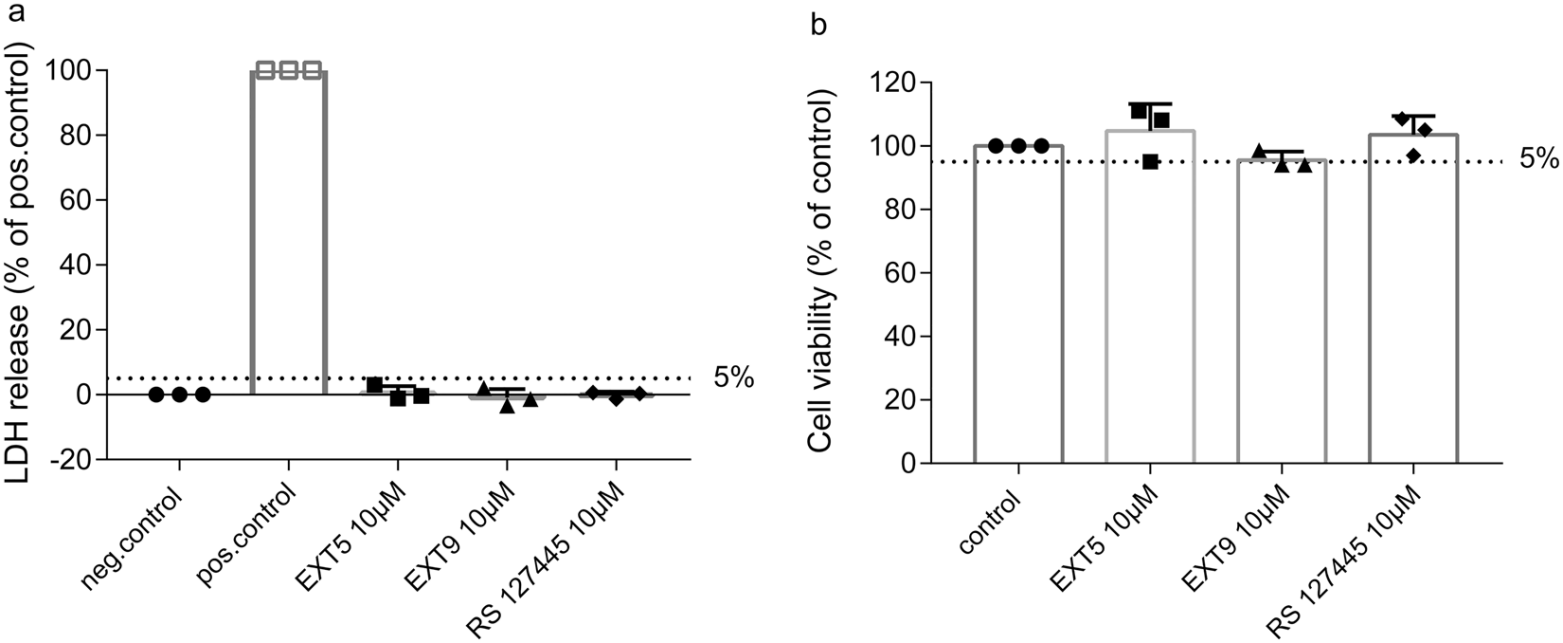
Measurement of cell mediated cytotoxicity in HFL-1 cells treated with 5-HT_2B_ receptor antagonist. HFL-1 cells, treated 48 h with 10 µM of EXT5, EXT9 or RS 127445, were examined for cell toxicity by measurement of LDH release (**a**) and cell viability (**b**). Untreated cells served as negative control (0%, **a**) or control (100%, **b**) for each assay. HFL-1 cells treated with 1% Triton-X100 served as positive control (**a**). A cut off, set to 5% from positive control (**a**) or control (**b**), was implemented for detection of cytotoxicity. Individual values are presented as mean + s.d., n = 3 independent experiments.

To exclude possible cytotoxic mediated effects, LDH release and cell viability were examined in human lung fibroblasts treated with 5-HT_2_ receptor antagonists. After 48 h exposure to 10 µM of EXT5, EXT9 or RS 127445, HFL-1 cells displayed neither elevated levels of LDH (Fig. 5a), nor a reduction in viability with WST-1 (Fig. 5b). Results show that none of the tested compounds at the highest tested concentration (10 µM) caused cell-mediated cytotoxicity, with the application of a 5% cut-off.

### Activated p21 pathway in human lung fibroblasts

**Figure 6:**
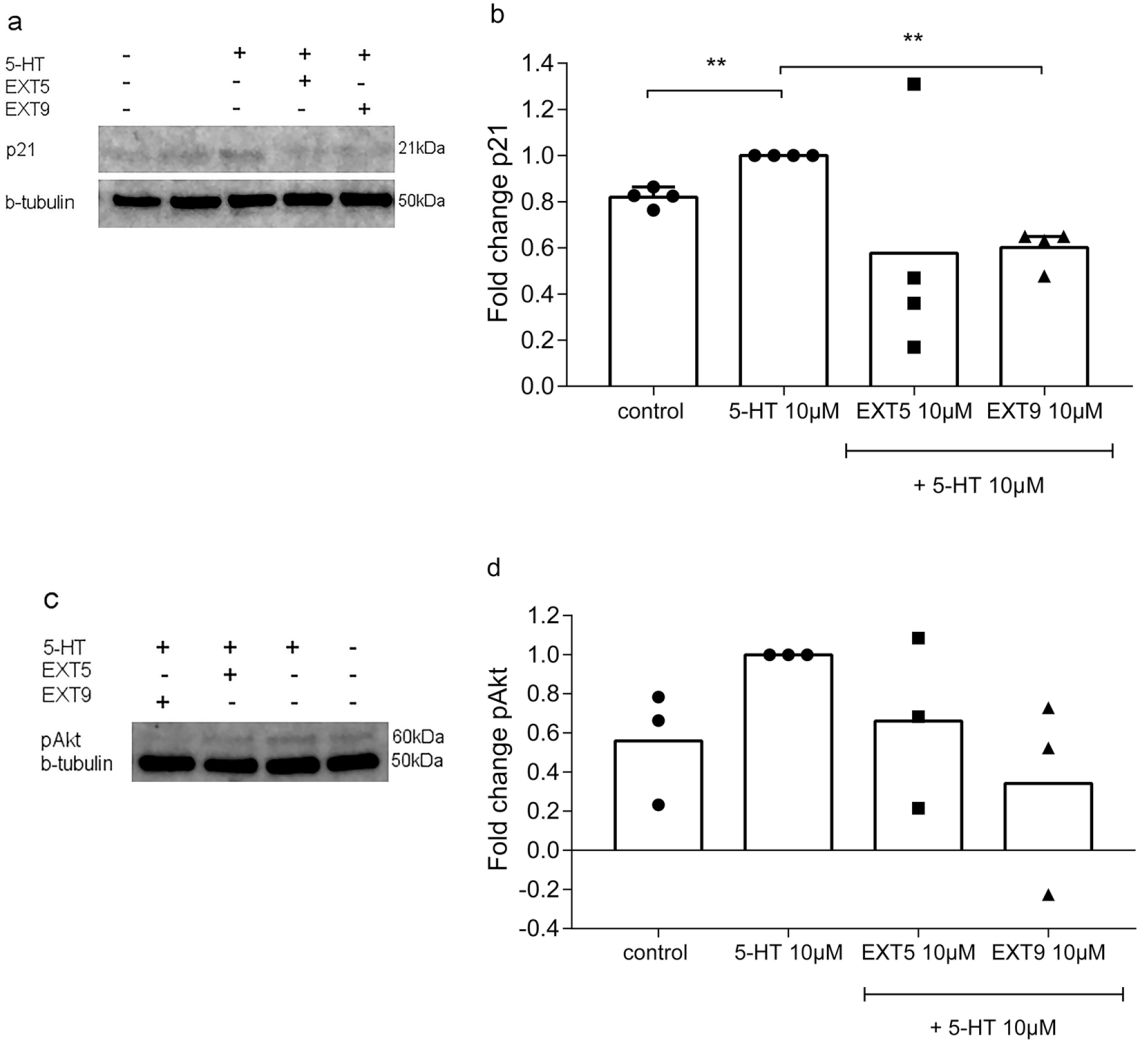
Protein levels of p21 and pAkt in HFL-1 cells treated with 5-HT and 5-HT_2B_ receptor antagonists. HFL-1 cells were pre-treated 1 h with or without EXT5 (10 µM) or EXT9 (10 µM). Next, cells were treated with 5-HT (10 µM) in combination with or without EXT5 and EXT9. Cell lysates were collected after 15 min and protein levels of p21, pAkt and the endogenous control β-tubulin were analyzed with western blot (**a**,**c**), with uniform arrangement of two separate protein bands as illustrated (**a**). Treatment with 0.1% DMSO served as control. Individual mean values are presented as fold change of p21 and pAkt in relation to 5-HT 10 µM, respectively. n = 3–4 (**b**,**d**).

The activation of the 5-HT_2B_ receptor triggers a signaling cascade resulting in the activation of the phosphatidylinositol-3 kinase, promoting the activation and phosphorylation of Akt^22^. To further examine 5-HT induced signaling pathways, levels of pAkt and the downstream signaling protein p21 were examined in human lung fibroblasts treated with 5-HT. HFL-1 cells display an increased protein level of p21 (FC = 0.18, p = 0.0032) and of pAkt (FC = 0.44, p = 0.12) following 15 min treatment with 5-HT (10 µM) (Fig. 6a–d). Treatment with the 5-HT_2B_ receptor antagonist EXT9 (10 µM) reduced levels of p21 (FC = 0.60, p = 0.0024) (Fig. 6b) and pAkt (FC = 0.34, p = 0.15) (Fig. 6d), in comparison to 5-HT alone (FC = 1). Similar effects were observed with EXT5; p21 (FC = 0.58) pAkt (FC = 0.66), although not proven statistical significant.

## Discussion

We have previously shown that an experimental induced model of pulmonary fibrosis, implementing systemic administrations of BLM, resulted in increased pulmonary tissue accompanied with increased amount of ECM-producing cells^4^. In this follow-up study, we identified an increased expression of *Cdkn1α*, a gene coding for the p21 protein, in BLM-treated mice. This protein, regulating cell growth was examined in human lung fibroblasts, serving as a translational study. Human lung fibroblasts demonstrated an induction of p21 following 5-HT-exposure, as well as the upstream signaling protein pAkt, which was reduced by treatment with 5-HT_2B_ receptor antagonists. Receptor mediated effects on cell proliferation and cell cycle entry showed a reduction in cellular growth, correlating to the recognized cell regulation pathways of pAkt/p21^12,18^.

Following intratracheal (local) BLM administration, severe damage occurs in epithelial cells, which is followed by activation and proliferation of fibroblasts, and subsequently also fibrosis^23^. Active and proliferative fibroblasts are thought to be key players in fibrosis, causing excess deposition of matrix proteins and collagen. Transforming growth factor (TGF) -β1, is a pro-fibrotic mediator stimulating proliferation and differentiation of fibroblasts into myofibroblast^24^ and upregulation of p21^25^; a marker for cellular damage and regulator in cell cycle progression^12^. In patients with IPF, p21 is overexpressed in terminal airways and alveoli^15^, highlighting distal lung regions of chronic cellular damage, which is the focus of the current study. Our previous studies have demonstrated that systemic administrations of BLM induced pulmonary fibrosis, with increased tissue density, as well as increased collagen staining (Masson’s trichrome and Picro Sirius red) and increased numbers of tissue myofibroblasts^3,4^. Systemic administrations of BLM initiates damage and stress to endothelial and epithelial cells, promoting the development of fibrosis in parallel with inflammation^3^. This mild to moderate model of pulmonary fibrosis, with low adverse effects, differs from the acute model of BLM-induced fibrosis using local administration. We thus wanted to investigate gene expression in distal lung tissue following systemic administration of BLM, seeking to identify signaling pathways involved in early fibroblast/myofibroblasts activation and proliferation. We identified *Cdkn1α* (gene coding for p21) to be significantly increased in BLM-treated mice in comparison to control animals (Table 1). These results supports previous findings in BLM-treated mice, which demonstrated an increased pulmonary expression of p21 positive cells^26^. A reduction in *Cdkn1α* with subsequently reduction of p21, may be indicative of a reduced cellular damage and less on-going repair process. Treatments with EXT5 or EXT9 showed tendencies in reducing the expression of *Cdkn1α* in comparison to BLM-treated mice, however, we were unable to confirm these results statistically. Comparisons of control animals to BLM-treated mice enable the identification of differential expressed genes, that showed tendencies in gene levels reverting back toward that of control animals following treatment with EXT, even though not significant. In our follow-up study examining both central and distal parts of the lung, similar expression patterns of *Cdkn1α* was seen in mice treated with EXT5 or EXT9, in comparison to BLM-treated mice. The heterogeneity of the disease model and usage of low systemic dosage of BLM may explain the small differences observed in between treatment groups. Origin of tissue is also crucial when studying this disorder, as the expression of *Cdkn1α* appears more prominent in parenchymal tissue. In summary, these two studies identify early changes in the gene expression of p21 in pulmonary fibrotic mice, with results indicative of 5-HT_2B_ receptor regulations.

The regulatory functions of p21 are highly complex, with several associated cellular mechanisms such as regulation of cell cycle progression and promotion of cell survival^17^. The nuclear export of cyclin D1 can also be inhibited by p21, thus promoting nuclear accumulation of cyclin D1, triggering cell cycling^27^. Cell cycle progression has been linked to 5-HT and 5-HT_2_ receptor activation^21,28^. In mouse fibroblasts transfected with the 5-HT_2B_ receptor, activation of receptors resulted in elevated cyclin D1 expression and thus increased cell proliferation, which was decreased with 5-HT_2B_ receptor antagonism^21^. In line with these findings, we showed that 5-HT enhances the entry of lung fibroblasts into the dividing phases of cell cycling, an event clearly diminished with 5-HT_2B_ receptor antagonism.

Interestingly, reduced levels of pulmonary pAkt, 5-HT and 5-HT_2B_ receptors have been associated with an attenuated inflammation and fibrosis^8^. In bovine pulmonary artery smooth muscle cells, 5-HT as well as a 5-HT_2_ agonist induced phosphorylation of Akt, an activation not hindered by the 5-HT_2B_ receptor antagonist - SB215505^28^. In this study we demonstrated anti-proliferative effects with 5-HT_2B_ receptor antagonists EXT5 and EXT9 in human lung fibroblasts, a non-toxic mechanism, previously shown to attenuate myofibroblast differentiation^4^. These results support a dual action of the 5-HT_2B_ receptor antagonists in inhibiting both cell differentiation and proliferation, however, distinguishing which cellular action is the foremost driving force in diminishing the development of fibrosis is still to be elucidated.

EXT5 and EXT9 have shown to display similar although varied effects in attenuating fibrosis *in vitro* and *in vivo*^4^, possibly explained by the compounds’ different receptor binding and functionality profiles. In current study, no distinct difference was detected in anti-fibrotic effects elicited by the two compounds, hence, the slight variance in receptor profiles appears less relevant.

We previously showed that EXT5 and EXT9 decreased the production of the proteoglycans such as decorin^4^. Interestingly, several studies have identified the interplay between p21 and decorin, presenting decorin as an inducer of p21^29,30^. In our previous study, we identified the upregulation of decorin in lungs of BLM-treated mice, as well as in human lung fibroblast treated with 5-HT in combination with TGF-β1^4^. Decorin production has shown to be increased in fibroblast clones from IPF patients, however, identified with a negative correlation between decorin synthesis and cell proliferation^31^. In current study, results showed the induction of p21 by 5-HT (10 µM), as well as the induction of pAkt, supporting previous findings in bovine smooth muscle cells^28^. The induction of pAkt and p21 may be linked to amplified levels of decorin via activation of 5-HT_2B_ receptors, a theory supported by reduced decorin production following treatment with EXT5 and EXT9^4^. However, with several anti-fibrotic effects documented, the cellular and molecular mechanisms of 5-HT_2B_ receptors antagonism in pulmonary fibrosis warrants further investigation^4,9^.

This study has focused on identifying early fibrotic events using a well characterized fetal cell line –a cell type linked to on-going regenerative processes in the development of fibrosis. In IPF patients^32^ and in BLM-induced fibrosis^9^ pulmonary expressions of 5-HT_2A_ and 5-HT_2B_ receptors are increased, whose expression on HFL-1 cells has been previously confirmed^4^. The anti-fibrotic effects mediated by EXT5 and EXT9 *in vivo*, translate to human cell culture systems, proposing the 5-HT_2B_ receptor antagonists as a novel approach in treating human pulmonary fibrotic disorders. For further clinical bearing, it would be of interest to examine a therapeutic treatment option with the compounds, when compound administration is applied later in the development of the disease. Studies have suggested 5-HT_2_ receptors as potential targets of novel treatment options for several fibrotic conditions such as hepatic fibrosis, systemic scleroderma and pulmonary hypertension^33^, thus highlighting the wide potential of selective 5-HT_2_ receptor antagonists in treating fibrosis of different tissue origin. In conclusion, the results from this study further identify the mechanism of 5-HT_2B_ receptors in associated pulmonary remodeling processes - affecting cell proliferation and associated signaling pathway pAkt/p21 both *in vitro* and *in vivo*.

## Methods

### Pulmonary fibrosis *in vivo* model

C57BL/6 mice (female, aged 12.5 weeks) (Scanbur research A/S, Karlslunde, Denmark) were injected s.c. with BLM three times/week for two weeks, with daily per oral (p.o.) treatment with EXT5 (30 mg/kg), EXT9 (30 mg/kg) or vehicle (Tween80, 2.5%w/v) as previously described^4^. The administration of compounds and vehicle were given simultaneously with first dose of BLM. Control animals were injected s.c. with saline, followed by p.o. treatment with vehicle. Seven animals were used per treatment/control groups and sacrificed 14 days after study initiation. In all treatment groups, animals displayed normal behavior, with an average weight loss of less than 3%. A shorter 10-day study in animals treated with or without the Tween80 vehicle (n = 5), showed no effect on behavior or weight loss (data not shown). Lungs were removed and immediately frozen on dry ice and stored in RNA-later. Study protocol was approved by the local ethics committee (Malmö/Lund, Sweden, M103-14), with methods performed in accordance with relevant guidelines and regulations. Receptor binding and functionality profile of receptor antagonists EXT5 and EXT9 (AnaMar AB, Lund, Sweden) presents slightly separated receptor binding and functionality profiles as previously stated^4^, with half maximal inhibitory concentration (IC_50_) of EXT5: 7.54 µM (5-HT_2A_), 0.082 µM (5-HT_2B_), 5.11 µM (5-HT_2C_); EXT9: 0.84 µM (5-HT_2A_), 0.029 µM (5-HT_2B_), 0.65 µM (5-HT_2C_).

### Whole genome gene expression analysis

Distal lung tissue was manually cut from lung lobes and homogenized with a rotator mixer in RLT buffer (Qiagen, Hilden, Germany) supplemented with β-mercaptoethanol. Samples were centrifuged at 5100xg for 3 min and supernatants were removed. RNA was extracted with RNeasy Mini Kit (Qiagen) according to manufacturer’s instructions, and measured with Nanophotometer (Implen GmbH, Munich, Germany). For RNA quality, samples were processed with Agilent RNA Nano 6000kit and Agilent 2100 Bioanalyzer (Agilent technologies, Santa Clara, CA, U.S.). RNA integrity number (RIN) was obtained with eukaryote total RNA nano assay. Gene expression data were collected with Illumina MousWG-6 v. 2 chips (Illumina Inc., Hayward, CA, U.S.). Data complying with MIAME standards are available in the NCBI GEO database under accession number GSE103511. For data N = 6 (N = number of animals analyzed per treatment group).

### RT-qPCR analysis

Murine lung tissues from the BLM-model were homogenized and extracted for RNA as previously described, with additional tissue homogenizing using QIA shredder (Qiagen, 79654). RNA samples were converted to cDNA with Reverse Transcriptase Kit (Cat.no. 205311, Qiagen) according to manufacturer’s instructions. cDNA samples were run in an rt-qPCR with PCR system Stratagene Mx3005P (Agilent Technologies, Santa Clara, CA, U.S.) using Quanti Tect SYBRGreen PCR kit (Cat.no. 204143, Qiagen) with probes for *Cdkn1α* (QT01752562, Qiagen) and *GAPDH* (QT01658692, Qiagen), according to manufacturer’s instructions. Values are normalized to reference gene *GAPDH* and the relative expression levels are determined by delta Ct values and average fold change.

### Cell proliferation assays

Human lung fibroblasts (HFL-1, CCL-153, ATCC, Manassas, VA, U.S.) (Caucasian, fetal cell line) were cultured in Dulbecco’s modified Eagle’s medium (DMEM, Sigma-Aldrich, St Louis, MO, U.S.), supplemented with 1% glutamine, 1% penicillin-streptomycin and 10% fetal clone serum (FCIII, Thermo Scientific, Waltham, MA, U.S.) at 37 °C, 10% CO_2_. Cells were seeded 5 000 cells/well in 96-well culture plates. After 6 h incubation, the cells were pre-treated 1 h with 5HT_2B_ receptor antagonists EXT5, EXT9 and RS 127445 (Tocris, Bristol, UK) (1 µM, 10 µM) (dissolved in DMSO) in DMEM medium with 1% serum. Cells were then treated with 5-HT (5-hydroxytryptamine hydrochloride, Sigma-Aldrich) (10 µM) in combination with 5-HT_2B_ receptor antagonists in 0.4% serum for 24 h. Cells were sub sequentially incubated with bromodeoxyuridine (BrdU) for additional 22 h ± 1 h. BrdU incorporation was measured using BrdU cell proliferation ELISA kit (ab126556, Abcam, Cambridge, U.K.), according to manufacturer’s instructions. In a similar methodological manner, cell amount of HFL-1 cells was quantified after 48 h incubation with 5-HT (10 µM) and 5-HT_2B_ receptor antagonist (10 µM) treatment in 0.4% serum, using no pre-treatment with receptor antagonists. Cells were fixed with glutaraldehyde 1% and stained with crystal violet 0.1% for 30 min^34^. After repeated washing steps in ddH_2_O, cells were treated overnight with 1% Triton-X100 and absorbance was quantified at 595 nm.

### Cell cycle assay

HFL-1 cells were seeded 400 000 cells in T25 cell culture flasks in supplemented DMEM with 10% FCIII. Cells incubated overnight at 37 °C, 10% CO_2_. Cells were pre-incubated with receptor antagonists (10 µM) in DMEM with 1% FCIII. The commercially available compound RS 127445 (5-HT_2B_ receptor antagonist) (Tocris, Bristol, UK) was used as a reference. After 1 h, cells were treated with 5-HT (10 µM) in combination with or without antagonists (10 µM). After 48 h incubation, cells were collected with trypsinization, counted and fixed for 30 min in ice cold 70% ethanol, at +4 °C. Cells were resuspended in D-PBS with 1% BSA and incubated with nuclear stain 7AAD (25 µg/ml, Sigma-Aldrich, A9400) and RNAseA (8 µg/ml, DNAse free, EN0531, Thermo Scientific) for minimum 10 min. Cells were acquired using flow cytometry in accuri C6 (BD Bioscience, San Jose, CA, U.S.) and analyzed with FlowJo LLC 10.3. Samples were run with slow flow rate with 20 000 events recorded per sample, with exclusion of doublets and debris by standard methodology. Linear format of FL3 were applied for detection of fluorescent emission per cell. Gating were set on 7AAD-peaks representing the standard G0/G1 and S, G2/M cell cycle phases. Data are presented as total cell count in S/G2/M phase in relation to all gated cells, shown in percent.

### Toxicity assays

HFL-1 cells were seeded at 5 000 cells/well in 96-well culture plates. After 6 h incubation, cells were stimulated with or without EXT5 (10 µM), EXT9 (10 µM) and RS127445 (10 µM) in 0.4% serum for 48 h. Cell medium was extracted and analyzed for lactate dehydrogenase (LDH) with cytotoxicity detection kit (Roche, Basel, Switzerland), according to manufacturer’s instructions. Cells treated with 1% Triton-X100 served as positive control. Cell viability was analyzed on adherent cells with tetrazolium salt WST-1 (diluted 1:10 in cell medium) (Roche). Cells were incubated at 37 °C, 10% CO_2_ and absorbance was quantified at 450 nm.

### Western blot

HFL-1 cells were seeded 250 000 cells/well in 6-well cell culture plates. After overnight incubation, cells were serum starved in DMEM medium with 1% serum. After 1 h, cells were treated with 5-HT (10 µM) in combination with 5-HT_2B_ receptor antagonists EXT5 (10 µM) or EXT9 (10 µM). Cell lysates were collected after 15 min in lysis buffer (NP-40, Sigma Aldrich) supplemented with cOmplete and PhosSTOP (Roche). Protein content was quantified with BCA assay kit (Thermo Scientific). With gel electrophoresis, proteins were size separated on Mini-Protean TGX stain free gel (Bio-Rad, Hercules, CA, U.S.) and transferred to a 0.2 µm PVDF membrane (Bio-Rad). Membrane was blocked and incubated with primary antibody (1:500, 1:1000) (90 min in RT, or overnight at 4 °C); anti-p21 (ab109199, Abcam), anti-phospho-Akt (Cell Signaling Technology, Danvers, MA, U.S.) and endogenous control β-tubulin (1:30 000) (ab6046, Abcam). Following washing steps in TBS-Tween 0.1%, membrane was incubated with secondary antibody DyLight800-conjugated anti-rabbit antibody (1:15 000). After final washing steps, core protein bands were visualized and quantified with Odyssey FC and Image Studio v. 5.2 (LI-COR Inc., Lincoln, NE, U.S.).

### Statistical analysis

Detection of differentially expressed genes was done by using significance analysis of microarrays (SAM) according to the instructions in the software manual (Tmev software (MeV v. 4.2)). The different treatments were all compared to control samples, and probes that were significant after correction for multiple testing were used to create Venn diagram and extract common patterns between the different treatments. Analysis was performed in R software (v. 3.3.1). With rt-qPCR, one-way ANOVA was used to analyze relative gene expression levels in between treatment groups. For *in vitro* results, one sample t-test was applied as commonly recommended when the numbers in groups are small, as a non-parametric tests based on ranks seldom demonstrate any differences^35^. Statistical software program Graph Pad Prism 7 (La Jolla, CA), were used for statistical calculations on results obtained *in vitro*. P-values of *p < 0.05, **p < 0.01 and ***p < 0.001 were considered as statistical significant.

### Data availability

The gene expression data that support the findings of this study are available from GEO data repository (NCBI) with further details described in Methods section. Remaining datasets generated during and/or analyzed during the current study are available from the corresponding author on reasonable request.
